# The relationship between parental involvement and psychological adjustment among Chinese children with autism spectrum disorder in the transition from kindergarten to primary school: A chain-mediating model

**DOI:** 10.3389/fpsyg.2023.1087729

**Published:** 2023-02-20

**Authors:** Yujia Hou, Tingrui Yan, Junfang Zhang

**Affiliations:** ^1^Early Childhood Education College, Shanghai Normal University, Shanghai, China; ^2^Special Education Department, Faculty of Education, East China Normal University, Shanghai, China; ^3^School of Special Education, Binzhou Medical University, Binzhou, Shandong, China

**Keywords:** parental involvement, psychological adjustment, parenting self-efficacy, parenting stress, transition

## Abstract

**Introduction:**

This study examined the impact of parental involvement on the psychological adjustment of children with autism spectrum disorder (ASD), and the role of parenting self-efficacy and parenting stress in the transition from kindergarten to primary school.

**Methods:**

Using the questionnaires, we collected data from 237 Chinese parents of children with ASD.

**Results:**

Mediation analyses showed that parental involvement partially promoted the psychological adjustment of children with ASD, which was reflected in the fact that parental involvement promoted children’s prosocial behavior but did not reduce their emotional/behavioral problems. Mediation analyses also revealed the role of the mediator in parenting stress between parental involvement and the psychological adjustment of children. Additionally, the results suggested that parenting self-efficacy and parenting stress played a chain-mediating role in the association between parental involvement and psychological adjustment in children with ASD.

**Discussion:**

These findings enhance our understanding of the mechanisms underlying the relationship between parental involvement and psychological adjustment in children with ASD in the transition from kindergarten to primary school.

## 1. Instruction

The transition from kindergarten to primary school has been regarded as a developmental and transformational process, marking a child’s beginning of formal schooling (Chan, 2010). For children with autism spectrum disorder (ASD), changes during the transition to primary school, including increased academic demands, reduced family support, changes in classroom routines, and new peer relationships to be established, may be overwhelming, influencing their psychological adjustment to enter primary school (Fontil et al., 2019; Nuske et al., 2019). Psychosocial adjustment reflects the abilities of an individual to adapt to the new surrounding environment and handle complex, significant, or critical challenges (Madariaga et al., 2014; Lan and Sun, 2022). Several studies have shown that 10–21% of children with and without developmental disabilities have difficulties in adjusting to primary school when they enter primary school, including school refusal, anxieties, temper tantrums, and even crying (Giallo et al., 2010; Heyne et al., 2017). Particularly for children with ASD, adaptation to a different environment, including a new role, new peer group, new teacher, and new expectations during the transition to primary school, may make them susceptible to mental health problems, especially maladjustment to the new school environment and social–emotional dysfunction (Di Biasi et al., 2016; Marsh et al., 2017). Some studies report that psychological maladjustment and mental health problems occur in children with ASD in the preschool phase, ranging from 30 to 60% (Lovell and Wetherell, 2016; Sha et al., 2022). Due to maladjustment, children with ASD are unable to effectively utilize emotion regulation strategies, and they demonstrate more depression and anxiety, resulting in lower achievement in primary school.

Social support theory suggests that parental involvement serves as a salient social resource to support children in addressing learning and social communication challenges, largely determining children’s developmental outcomes (Sendra et al., 2020). Parental involvement includes a series of activities conducted by parents at home and school to enhance their children’s academic and social development (Seginer, 2006). Parental involvement has been shown to be related to improved social skills, less loneliness and anxiety, and positive peer relationships, which are necessary for children to improve psychological adaptation and successfully achieve the transition (Garbacz et al., 2018). In the transition phase, parents can foster emotional adjustment and alleviate psychological anxiety in their children through home-based guidance and education, thus improving their psychological adjustment. Considering the benefit of parental involvement on children’s transition, *The Guidelines on Promoting the Scientific Transition from Kindergarten to Primary School* was issued by Ministry of Education of China (2021), in which parental involvement was considered as one of the best practice strategies for achieving smooth transition (Ministry of Education of China, 2021; Hou et al., 2022). For children with ASD, parental involvement promotes their cognitive and academic development and effectively reduces their emotional and behavioral problems caused by maladjustment to the changes that occur during the transition to primary school (Dockett and Perry, 2007; Schiltz et al., 2018).

Given the lag in language development and the deficit in the social communication of children with ASD, parental involvement in transition improves their psychological adjustment to address problems and challenges in school readiness and adaption (Wang et al., 2020). However, although extant research has focused largely on parental involvement and children’s school adjustment in the transition to primary school on typically developing children in China (Chen et al., 2020; Lau, 2014; Xia et al., 2020), studies on samples of children with ASD are ignored. As resistance to change, one of the core symptoms of ASD, makes the transition to primary school even more challenging for children with ASD, it is urgent to examine how parental involvement can improve psychological adjustment in children with ASD. Furthermore, most existing studies have focused on the direct relationships between parental involvement and children’s academic development and school readiness (Chang et al., 2009; Karbach et al., 2013). Little is known about the mechanisms underlying parental involvement and psychological adjustment in children with ASD. As a parenting behavior, the frequency and quality of parental involvement may affect the psychological status of parents, such as parenting self-efficacy and parenting stress, which further affects the psychological adjustment of children with ASD during the transition to primary school. Hence, this study mainly focused on the cognitive behavioral mechanisms underlying the association between parental involvement and psychological adjustment in children with ASD during the transition from kindergarten to primary school.

Parenting self-efficacy reflects parents’ perceptions of their competence in parenting (Jones and Prinz, 2005; Gavita et al., 2014). Most researchers have reported that parents’ parenting self-efficacy largely determines their parenting behaviors and is closely linked to their children’s psychosocial adjustment and social–emotional development (Junttila et al., 2007; Trecca et al., 2022). Roskam et al. (2015) found that high parenting self-efficacy could effectively reduce children’s aggressive and disobedient behavior. Children nurtured by parents with high parenting self-efficacy directly demonstrate a high level of interpersonal communication skills and show more prosocial behaviors, such as empathy, caring for others, attention, and listening (Luengo et al., 2021). During the critical period of transition to primary school, the more confident parents are in their competence to develop children’s school readiness and school adaptation, the more likely they are to employ positive parenting strategies to address parenting challenges, and finally promote smooth and positive transition outcomes for children (Giallo et al., 2008). However, despite numerous studies on parents’ psychological well-being and positive parenting (Pastorelli et al., 2016; Li et al., 2021), no investigation has yet reported on the effect of parenting self-efficacy on psychosocial adjustment of children with ASD during the transition from kindergarten to school.

While parental involvement and self-efficacy can improve the psychological adjustment of children with ASD, this positive effect may be weakened and inhibited by the continued accumulation of parenting stress (Holloway et al., 2016; Silinskas et al., 2020). Parenting stress refers to negative psychological experiences, such as anxiety, depression, and self-reproach, associated with the fulfillment of parents’ roles (Abidin, 1995; McStay et al., 2014). Several studies have reported that parents of children with ASD suffer from more stress than parents of children without disabilities (Hutchison et al., 2016; Yamane, 2021). Furthermore, the stress of raising a child with ASD may be fundamentally different from that experienced by parents of children without disabilities (Lu et al., 2018; Stephenson et al., 2022). Parenting stress in parents of children with ASD generally leads to less sensitive and responsive parenting and more behavioral problems for their children (Lickenbrock et al., 2011). In particular, many Chinese parents worry that their children with ASD have internal emotional disorders, external behavioral problems, and lower academic development due to maladjustment to primary school, which puts parents under great stress (Wang et al., 2020). Moreover, parenting stress has also been reported to have a negative effect on social–psychological adaption in children and can positively predict their internalizing behavioral problems (Albanese et al., 2019; Kochanova et al., 2021). Some studies on families of children with ASD have suggested that maternal parenting stress predicts children’s social cognition deficiency, poor language power of expression, weak emotion regulation ability, and lack of social skills (Hayes and Watson, 2013).

In addition, according to the parenting stress model, poor interaction between parents and children can lead to higher parenting stress which also affect children’s social communication (Estes et al., 2009; Qian et al., 2022). Hence, low levels of parental involvement in transition activities may increase parental parenting stress, thus hindering children’s social adjustment (Rezendes and Scarpa, 2011). Some studies have found that maternal parenting stress serves as the mediation variable between paternal involvement and children’s social adaptability, in which high level of parental involvement can alleviate parenting stress and, in turn, reduce internalized behavioral problems of withdrawn children (Luo et al., 2020). Therefore, parenting stress could play a mediating role in the psychological adjustment of children with ASD during the transition to primary school through parental involvement.

Mowder’s (2005) parent development theory proposed that as parents interacted more with their children, their perceptions of parenting were modified by parenting behavior over time. As the transition to primary school is a crucial period for parents to develop their sense of competency and confidence—and as parents are also vulnerable to mental health problems—it is pressing to reveal the mechanism by which parental involvement influences the psychological adjustment of children with ASD through parenting psychology. However, although several studies have separately examined the role of parenting self-efficacy and parenting stress as mediators between parental involvement and children’s psychological adjustment (Semke et al., 2010; Li and Wei, 2017), few studies have included these two parenting psychological variables into a comprehensive effect model of parental involvement on children’s psychological adjustment. Previous research has suggested that higher parenting self-efficacy predicts lower levels of parenting stress and enhanced social adjustment in children with ASD (May et al., 2015; Chan et al., 2021). For example, Keen et al. (2010) found that among parents of children with ASD, interventions aimed at improving parenting self-efficacy could effectively reduce parenting stress. Other studies have also revealed that higher parenting self-efficacy is related to lower levels of stress, whereas lower efficacy predicts more stress (Bloomfield and Kendall, 2012). It could be inferred from existing studies that parenting self-efficacy and parenting stress may potentially serve as mediating functions between parental involvement and psychological adjustment. Therefore, two variables—parenting stress and parenting self-efficacy—were included in the association between parental involvement and children’s psychological adjustment in the current study.

## 2. The current study

The importance of parental involvement in the transition to primary school in children’s psychological adjustment has been highlighted in a wealth of research (Fan and Chen, 2001; Geenen et al., 2001). However, the mechanism underlying the effect of parental involvement on psychological adjustment in children with ASD remains unclear. By investigating parents of children with ASD, we aimed to explore the effect of parental involvement on psychological adjustment and the chain mediation role of parenting self-efficacy and parenting stress in the Chinese context. The chain mediation means that the independent variable affects the dependent variable through at least two mediating variables in the sequential order. The research hypotheses are as follows:

*H1*: Higher parental involvement is associated with higher psychological adjustment in children with ASD.

*H2*: Parenting self-efficacy mediates the relationship between parental involvement and psychological adjustment in children with ASD.

*H3*: Parenting stress mediates the relationship between parental involvement and psychological adjustment in children with ASD.

*H4*: There is a chain-mediating mechanism in which parenting stress and self-efficacy sequentially mediate the relationship between parental involvement and psychological adjustment in children with ASD.

## 3. Method

### 3.1. Participants

Participants in the current study were recruited from several Chinese cities, including Beijing, Zhengzhou, Weifang, and Xiamen. The inclusion criteria of participants were as follows: (a) parents served as the primary caregivers for their children in the family; (b) their children were diagnosed with ASD by qualified health professionals who are empowered by Chinese health authorities; and (c) their children with ASD were getting ready for primary school or just entering primary school. Moreover, to ensure the authenticity of the sample, we set exclusionary criteria, excluding parents whose children had multiple disorders, such as both ASD and intellectual disability, both ASD and attention deficit hyperactivity disorder (ADHD), or other diseases. In total, 245 eligible parents participated in the study and completed questionnaires. Of the 245 returned questionnaires, eight were excluded due to incomplete data, leaving 237 valid questionnaires for formal data analysis. The average age of the participants, 183 mothers and 54 fathers, was 33.3 years (SD = 6.04). The average age of children with ASD, including 163 boys (68.8%) and 74 girls (31.2%), was 7.6 years (SD = 1.07). Table 1 shows the demographics of parents and their children with ASD.

**Table 1:** The demographics of the participants.

Characteristics	Mean (SD)	*n*	%
Children’s age	7.6 (1.07)		
Children’s gender			
Male		163	68.8
Female		74	31.2
Parents’ gender			
Male		54	22.8
Female		183	77.2
Parents’ age	33.3 (6.04)		
Educational level			
Junior high school and below		89	37.5
High school diploma		57	24.1
Junior college degree		36	15.2
Bachelors		35	14.7
Masters and above		20	8.4
Household income			
Below ¥4,000		88	37.1
¥4,000–¥6,000		63	26.6
¥6,000–¥8,000		25	10.5
¥8,000–¥10,000		29	12.2
Above ¥10,000		32	13.5

### 3.2. Measures

#### 3.2.1. Scale of parental involvement in transition from kindergarten to primary school

Parental involvement was measured using the Parental Involvement in Transition from Kindergarten to Primary School Scale (PITKPS), an assessment tool developed specifically for children with developmental disabilities in China by Hou (2021). The 35-item measure included six dimensions: cognitive guidance, skill, emotional support, community connection, school choice decisions, communication and consultation, and self-learning and reflection. With self-reporting, parents rated their involvement in the transition to primary school on a five-point Likert-type scale (1 = completely disagree, 5 = completely agree). The Cronbach’s alpha coefficient of the PITKPS was 0.923 in the current study, which exhibited high internal reliability. The validity of the scale was assessed in the current study, *χ*^2^/df = 2.06, NFI =0.94, CFI = 0.93, RMSEA = 0.05, suggesting a good model fit of six factors.

#### 3.2.2. Parenting sense of competence scale

The Chinese version of the Parenting Sense of Competence (PSOC) Scale adapted by Peng et al. (2012) was utilized to assess parenting self-efficacy. The PSOC is a self-reported measure consisting of 17 items from the efficacy and satisfaction subscales. Only the 12-item efficacy subscale was used in the present study. Participants responded to the item on a four-point Likert scale, with scores ranging from 1 (strongly disagree) to 4 (strongly agree). With a Cronbach’s alpha coefficient of 0.825, the efficacy subscale of the PSOC showed good internal consistency reliability. The validity analysis results of the scale showed a good model fit, *χ*^2^/df = 2.14, RMSEA = 0.07, NFI = 0.93, and CFI = 0.92.

#### 3.2.3. Parental stress scale

Parental stress was measured using the 14-item Parental Stress Scale (PSS) revised by Zhang (2017). Parents assessed their stress levels on a five-point Likert-type scale, with higher scores indicating higher levels of parenting stress for parents of children with ASD. The Cronbach’s alpha coefficient of this scale is 0.846, indicating a higher internal consistency in the present study. For the validity of the scale, the results showed that *χ*^2^/df was 3.25, RMSEA was 0.09, NFI was 0.90, and CFI was 0.91, which meant a reasonable model fit.

#### 3.2.4. Strengths and difficulties questionnaire

The Chinese version of the Strengths and Difficulties Questionnaire (SDQ) adapted by Kou et al. (2005) from Goodman (2001) was used to measure the psychological adjustment of children with ASD. The SDQ is a parent-reported measure that contains 25 items from five subscales: emotional symptoms, hyperactivity, peer problems, conduct problems, and prosocial behavior (Haynes et al., 2013). The first four dimensions were used to measure emotional and behavioral problems in children with ASD, representing their difficult behavior. The fifth dimension, prosocial behavior, represented children’s strength. The SDQ was completed by parents of children with ASD using a three-point Likert scale ranging from 0 (“not true”) to 2 (“certainly true”) (Karst and Van Hecke, 2012). Both a higher score of prosocial behavior and a lower score of emotional/behavioral problems (the total score for the first four dimensions) indicated higher psychological adjustment in children with ASD. The SDQ has been widely used in previous studies to assess the psychological adjustment of children (Rodrigues et al., 2019; Teuber et al., 2022). Cronbach’s alpha for the strengths and difficulties subscales of the SDQ was 0.872 and 0.911, respectively, in the current study. The validity analysis of the scale reported a reasonable model fit, *χ*^2^/df = 3.41, RMSEA = 0.08, NFI = 0.91, and CFI = 0.92.

### 3.3. Procedure

Parents of children with ASD from Beijing, Guangzhou, Chongqing, Zhengzhou, Lanzhou, and Wuhan were first selected using convenience sampling (Li et al., 2022). We recruited 258 participants who were willing to participate in the research project by contacting many school principals and kindergarten principals who provided support for this study. Based on the inclusion criteria described above, 13 parents were excluded because their children had reached third grade and above, leaving 245 eligible parents. To ensure participant autonomy and privacy rights, we explained to the participants the purpose and meaning of the study, the potential effects of the study on their children, and the confidentiality of the data. Subsequently, written informed consent forms and questionnaires were distributed to the participants and collected on-site. After the initial screening, nine questionnaires were eliminated because many items were left unanswered. Finally, 237 valid questionnaires were entered into the formal data processing procedures.

### 3.4. Data analysis

First, although the scales used in this study were all mature scales that have been widely used, the reliability and validity of the scales were confirmed. Specifically, the reliability of each scale was analyzed by SPSS software. Then the confirmatory factor analysis (CFA) was used to assess the validity of each scale by Amos. Second, we conducted a correlation analysis between the demographic variables and the key five variables (parental involvement, parenting self-efficacy, parenting stress, emotional/behavioral problems and prosocial behaviors) to determine the control variables in the next analysis. Next, the Pearson correlation was performed to determine the relationships between the key variables, which is the prerequisite for mediation analysis. Then, to verify hypothesis 1, the hierarchy regression analysis for the emotional/behavioral problems and prosocial behaviors were conducted to determine the inferential relationship according to the sample data in this study.

Third, to verify Hypotheses 2–4, mediation analysis was used to examine the chain mediated effects of parenting self-efficacy and parenting stress between parental involvement and psychological adjustment in a holistic model, which had been widely used previously (Beeble et al., 2009; Parkes and Sweeting, 2018). We characterized a significant mediated effect of parenting self-efficacy or parenting stress between parental involvement and psychological adjustment if the effect of the independent variable on the dependent variable was mediated by one mediating variable; a significant chain mediated effect if the effect of the independent variable on the dependent variable was mediated sequentially by two mediating variables.

Mediation analysis was performed with PROCESS in SPSS. As emotional/behavioral problems and prosocial behaviors represent two opposing aspects of psychological adjustment in children with ASD, two models were used to examine the effect of parental involvement on children’s psychological adjustment. In these two models, parental involvement was incorporated as the independent variable (*X*), and parenting self-efficacy and parenting stress served as the first-order mediator (*M*1) and second-order mediator (*M*2), respectively. Emotional/behavioral problems and prosocial behavior in children with ASD were the dependent variables (*Y*) (Lu et al., 2021). To determine the significance of mediation, we also used a bias-corrected bootstrap estimation approach with 5,000 samples in the study. A 95% confidence interval (CI) does not include zero, indicating that the mediating effect is significant (Yuan and Hayashi, 2003).

## 4. Results

### 4.1. Descriptive statistics, correlations, and regression

First, correlation analysis and ANOVA were used to check whether there were significant differences between the five main variables and demographic variables. The preliminary results showed that parents’ age was negatively linked with parental involvement (*r* = −0.202, *p* < 0.01); meanwhile, parenting self-efficacy varied significantly by household income (*F*_(4,237)_ = 3.747, *p* < 0.01, *η*^2^ = 0.075). Thus, the age of the parents and household income were included as covariates.

Table 2 shows the means and standard deviations of all the main variables (parental involvement, parenting efficacy, parenting stress, emotional/behavioral problems, and prosocial behavior representing psychological adjustment) in the measures. Considering that the correlation between key variables was a prerequisite for mediation analysis, Pearson’s correlations between parental involvement, parenting stress, parenting self-efficacy, emotional/behavioral problems, and prosocial behavior were calculated separately. Table 2 presents the significant intercorrelations between parental involvement, parenting stress, parenting self-efficacy, emotional/behavioral problems, and prosocial behavior. The correlation analysis showed a significant positive correlation between parental involvement and parenting self-efficacy, a significant negative relationship between parenting self-efficacy and parenting stress, and the significant relationships among parenting self-efficacy, parenting stress and the two dimensions of psychological adjustment.

**Table 2:** Mean scores for parental involvement, parenting self-efficacy, parenting stress, emotional/behavioral problem, and prosocial behavior and summary of Pearson correlations between these variables.

	*M*	*SD*	1	2	3	4	5
1. Parental involvement	3.41	0.73	1				
2. parenting self-efficacy	2.87	0.47	0.587***	1			
3. Parenting stress	2.84	0.58	−0.361***	−0.346***			
4. Emotional /behavioral problems	2.05	0.53	0.520***	−0.322***	0.324***	1	
5. Prosocial behavior	1.77	0.25	−0.143*	0.370***	−0.123*	−0.350***	-

*p < 0.05; **p < 0.01; ***p < 0.001.

Additionally, the hierarchical regression analyses were also performed to examine the predictive effect of parental involvement on psychological adjustment of children with ASD (see Table 3). It was demonstrated that the estimated coefficient of parental involvement was significantly positive at the 1% level in the regressions for prosocial behavior, suggesting that the higher the level of parental involvement, the more the prosocial behaviors. Moreover, the regression analysis also showed that the estimated coefficient of parental involvement was not significant at the 5% level in the regressions for emotional/behavioral problems. These results partially verified Hypotheses 1.

**Table 3:** Relationship between parental involvement and psychological adjustment in children with ASD.

Dependent variable	Prosocial behavior			Emotional/Behavioral problems		
	*B*	*SE*	*β*	*B*	*SE*	*β*
Step1						
Parents’ age	−0.010	0.006	−0.114	−0.001	0.003	−0.032
House income	−0.050	0.022	−0.145*	−0.003	0.010	−0.20
Step 2						
Parenting stress	−0.227	0.058	−0.249***	0.158	0.028	0.375***
parenting self-efficacy	0.265	0.071	0.237***	0.002	0.034	0.004
Step 3						
Parental involvement	0.338	0.050	0.460***	−0.013	0.026	−0.037

*p < 0.05; **p < 0.01; ***p < 0.001.

### 4.2. Chain mediation analysis

**Figure 1:**
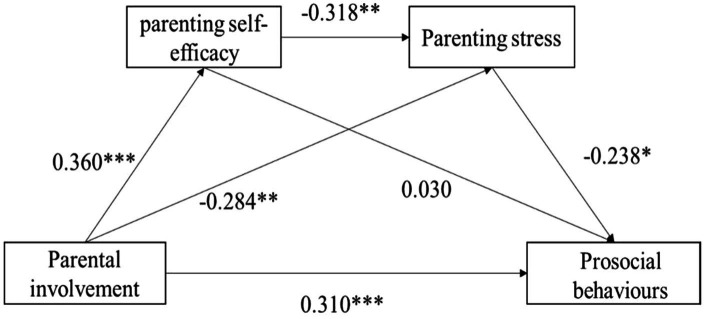
Mediation model testing the association between parental involvement and prosocial behaviors *via* parenting self-efficacy and parenting stress. *p < 0.05; **p < 0.01; ***p < 0.001.

Figure 1 shows the analysis results of the mediating effect of parenting stress and parenting self-efficacy between parental involvement and prosocial behavior (PROCESS, Model 6). After controlling for the effect of parents’ age and household income, the regression coefficient of each path was significant, except for the path from parenting self-efficacy to prosocial behavior (see Table 4). When parenting self-efficacy and parenting stress were included, the direct effect of parental involvement on children’s prosocial behavior was significant (*β* = 0.310, *SE* = 0.049, 95% CI (0.300, 0.456)). The indirect effect of parental involvement on parenting self-efficacy and parenting stress was significant [*β* = 0.027, *SE* = 0.007, 95% CI (0.002, 0.033)]. Additionally, the mediation path of parental involvement to prosocial behavior through parenting stress was significant [*β* = 0.068, *SE* = 0.014, 95% CI (0.006, 0.062)].

**Table 4:** Standardized indirect effects and 95% CIs for the mediational model.

Pathway	Effect	*SE*	LLCI	ULCI
Parental involvement—Prosocial behavior	0.310	0.049	0.300	0.456
Parental involvement—parenting self-efficacy —Prosocial behavior	0.011	0.0287	−0.050	0.062
Parental involvement—parenting self-efficacy —Parenting stress—Prosocial behavior	0.027	0.007	0.002	0.033
Parental involvement—Parenting stress—Prosocial behavior	0.068	0.014	0.006	0.062

**Figure 2:**
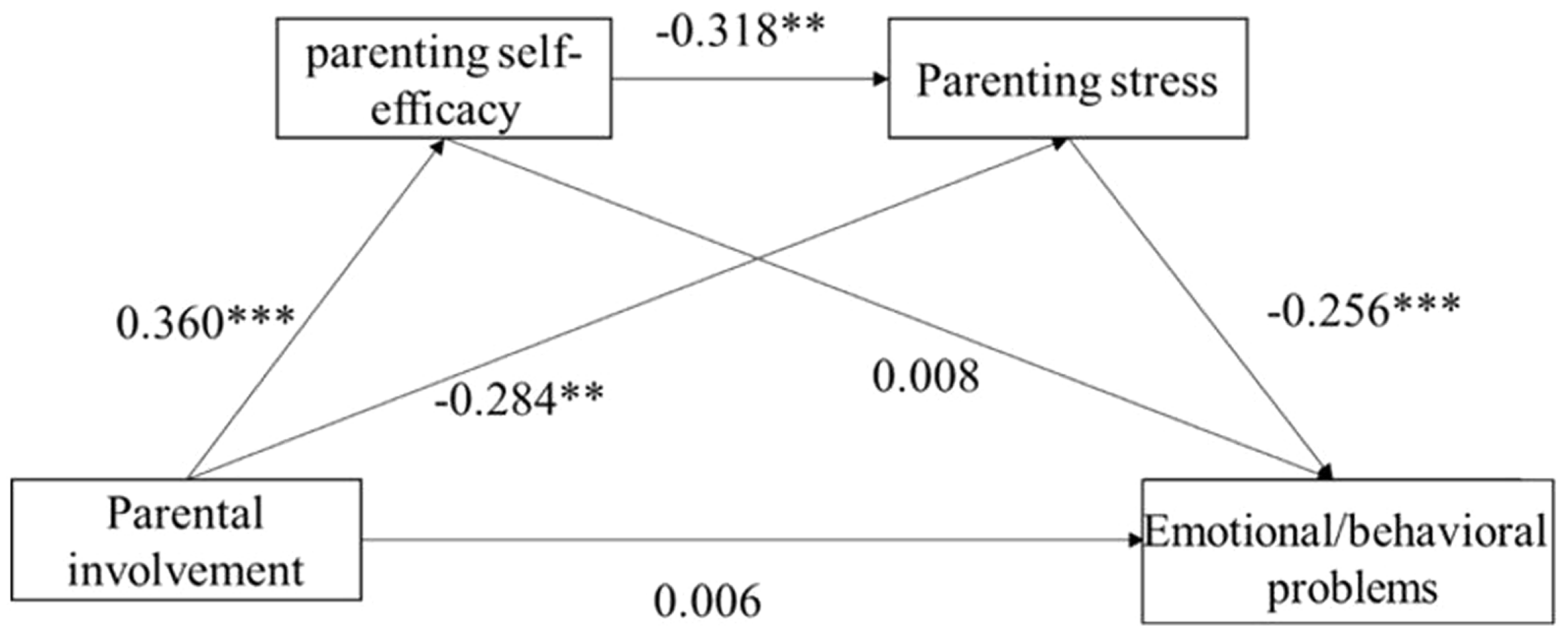
Mediation model testing the association between parental involvement and children’s emotional/behavioral problems *via* parenting self-efficacy and parenting stress. *p < 0.05; **p < 0.01; ***p < 0.001.

Next, we examined whether parenting self-efficacy and parenting stress mediated the relationship between parental involvement and emotional/behavioral problems. Model 6 in PROCESS for SPSS 24.0 was performed as above, with the effect of parents’ age and household income controlled (see Figure 2). As shown in Table 5, the direct effect of parental involvement on children’s emotional/behavioral problems was not significant [*β* = −0.006, SE = 0.025, 95% CI (−0.056, 0.043)]. However, the indirect effect of parental involvement on children’s emotional/behavioral problems through parenting self-efficacy and parenting stress was significant [*β* = −0.029, SE = 0.007, 95% CI (−0.031, −0.003)]. Our analysis also showed that the mediation path of parental involvement to children’s emotional/behavioral problems through parenting stress was significant [*β* = −0.073, SE = 0.011, 95% CI (−0.058, −0.012)]. The mediation path of parental involvement in children’s emotional/behavioral problems through parenting self-efficacy was not significant [*β* = 0.003, SE = 0.016, 95% CI (−0.030, 0.033)].

**Table 5:** Standardized indirect effects and 95% CIs for the mediational model.

Pathway	Effect	*SE*	LLCI	ULCI
Parental involvement—emotional/behaviral problems	−0.006	0.025	−0.056	0.043
Parental involvement—parenting self-efficacy —emotional/behaviral problems	0.003	0.016	−0.030	0.033
Parental involvement—parenting self-efficacy —Parenting stress—emotional/behaviral problems	−0.029	0.007	−0.031	−0.003
Parental involvement—Parenting stress—emotional/behaviral problems	−0.073	0.011	−0.058	−0.012

## 5. Discussion

Parents can help children with ASD improve their psychological adjustment, which is irreplaceable in achieving a smooth transition from kindergarten to primary school (Besi and Sakellariou, 2019; Hou et al., 2022). This study was designed to examine the mechanisms by which parental involvement affects the psychological adjustment of children with ASD during their transition to primary school. The strength of this study is that it was done in a Chinese sample, where there has been limited research on the relationship between parental involvement, parenting self-efficacy, parenting stress, and children’s psychological adjustment, and how culture may affect some of these constructs in this sample. This study further deepens our understanding of the different pathways from parental involvement to the psychological adjustment in children with ASD, including prosocial behavior and emotional/behavioral problems, by operating in a different way in the Chinese social context.

The present study showed that parental involvement directly promoted children’s prosocial behavior, but did not directly reduce the emotional/behavioral problems that occurred during the transition to primary school, which is partially consistent with our hypothesis. The positive relationship between parental involvement and prosocial behavior in children with ASD suggests that children with ASD would demonstrate more positive behaviors such as helping others, sharing, cooperating, and being comfortable if parents were more involved in preparing their children for school. The findings provide evidence for social support theory, proposing that parental involvement functions as an essential social resource to determine positive developmental outcomes of children (Cohen and Wills, 1985; Lau and Power, 2018). Specifically, when children with ASD are preparing for or just entering primary school, the more the parents engage in language and cognitive activities at home and communicate with the teachers, the better their children’s prosocial behavior (Lau and Power, 2018). These findings support previous studies reporting that highly involved parents are more likely to nurture their children’s helping behaviors and social–emotional traits (e.g., empathy and perspective-taking; Carlo et al., 2018; Gülseven and Carlo, 2021).

In addition, the study also found that while parental involvement was marginally related to emotional/behavioral problems in children with ASD, it did not directly reduce them during the transition to a new educational setting. This is in accordance with prior research finding that maternal home-based involvement does not directly reduce the social dysfunction of children with ASD unless family cultural capital is high (Yan and Hou, 2022). Therefore, it is believed that parental involvement in parent–child activities and cognitive guidance activities in kindergarten predicts children’s positive outcomes but does not directly reduce the negative aspects of children during the transition (Powell et al., 2012; Lau and Power, 2018).

The role of parenting stress as a mediator between parental involvement and children’s psychological adjustment during the transition, including prosocial behavior and emotional/behavioral problems, was found in the current study. This supports previous studies suggesting that parental involvement can reduce parenting stress and, in turn, increase children’s prosocial behavior and decrease their emotional/behavioral problems (Blake Berryhill, 2018; Williams et al., 2020). Parenting stress has been linked to delayed social competence and increased internalizing problems in children (Anthony et al., 2005; Calkins and Perry, 2016). The parenting stress that parents experience in improving children’s school readiness and school adjustment is transferred to parental participation actions to weaken the effect of positive parenting behavior, thus hindering children’s mental health development during the transition to primary school (Bakker and Demerouti, 2013; Hou et al., 2022). Hence, the boosting effect of parental involvement as positive parenting on the psychological adjustment of children is largely dependent on the reduction of parenting stress. For example, Williams et al. (2020) found that compared with uninvolved parents, highly involved parents experienced constantly decreasing parenting stress; toddlers raised by highly involved parents exhibited increased communicative behavior and greater improvements in interactions with other adults and peers (Williams et al., 2020).

The present study provides evidence that parenting self-efficacy and parenting stress play a chain-mediating role in the relationship between parental involvement and psychological adjustment in children with ASD. This finding echoes a two-phase transformation framework on parental involvement among children with developmental disabilities proposed by Hou et al. (2022), in which as parental involvement progressed from aggressive to rational involvement, Chinese parents of children with developmental disabilities gradually improved their parenting self-efficacy through self-reflection and social support, thus decreasing their parenting stress and finally promoting their children’s transition outcome (Hou et al., 2022).

The increase in parental involvement in family education and home–school communication experience has an obvious effect on their parenting efficacy (Benson, 2015; Peng and Li, 2020). As involved parents communicate and interact more with their children in transition activities, their feelings of competence and self-efficacy are also enhanced, leading to a reduction in stress over time. However, parenting self-efficacy, as a positive emotional experience of parenting, can inhibit the adverse impact of parenting stress on the adaptive behavior of children with ASD. Parents with high self-efficacy tend to employ positive psychological strategies, such as maintaining an optimistic attitude and an appropriate toughness, to deal with stressful problems and challenging tasks (Chan et al., 2021). Several studies have also revealed a strong negative correlation between parenting self-efficacy and parenting stress, indicating that higher parenting self-efficacy is crucial for decreasing parenting stress (Hassall et al., 2005). Therefore, parenting self-efficacy and parenting stress sequentially bridge the association between parental involvement and psychological adjustment in children with ASD.

## 6. Conclusion and limitations

From a family perspective, the current study focused on the transition to primary school for children with ASD by examining the psychological and behavioral characteristics of the parents. In conclusion, although prior research has recognized the benefits of parental involvement in children with ASD on psychological adjustment (Chan et al., 2021), our findings provided evidence that the relationship between parental involvement and psychological adjustment in children with ASD was mediated sequentially by parenting self-efficacy and parenting stress. More importantly, we also concluded that parenting self-efficacy and parenting stress operate differently in this process. The mediation mechanism by which parental involvement affects the psychological adjustment of children with ASD goes beyond our traditional understanding, suggesting the importance of parental psychological resources and the benefits of promoting parental involvement in children’s adjustment to new formal education during the transitional period.

More importantly, this research has some potential implications for future practices aimed at improving the psychological adjustment in children with ASD. First, given the specific nature of the transition from kindergarten to primary school, parents can participate in their child’s transition activities in more ways than one, including cognitive guidance, skill and emotional support, community connection, school choice decisions, communication and consultation, and self-learning and reflection. These supports in terms of parental involvement go beyond academic support and social development support, and also include the social support that parents strive for their children with ASD. Second, with the mediation role of parenting stress and parenting self-efficacy, targeted measures including individual and family interventions, community interventions, and service-related interventions should be taken to reduce parenting stress (e.g., anxiety, depression) and increase parenting self-efficacy in parents who raise children with ASD. Some evidenced-based practices, such as Mindfulness-based interventions, Acceptance and Commitment Therapy (ACT), and Emotionally-Focused Therapy (EFT), should be adopted to teach parents to cope with negative experiences and enhance their self-efficacy (Lee et al., 2017; Rayan and Ahmad, 2017; Lunsky et al., 2018).

Despite these advantages, several limitations of these results should not be ignored. First, the use of self-reported measures in this study may have resulted in unfaithful responses from parents of children with ASD. Despite the researchers’ assurances during data collection that their answers would remain confidential, participants might still have doubts and concerns about expressing their own high parenting stress and the low psychological adjustment of their children. Therefore, alternative data collection strategies, such as a combination of parental and teacher reports, may need to be employed in future studies to address these measurement errors. Second, fathers were recruited as much as possible, but most participants in the current study were mothers. While this guarantees information from the child’s primary caregiver, it can also lead to a gender imbalance in the sample.

## Data availability statement

The original data in the study is provided on demand. Further inquiries can be directed to the corresponding author.

## Ethics statement

The studies involving human participants were reviewed and approved by East China Normal University. The participants provided their written informed consent to participate in this study.

## Author contributions

YH was responsible for the data analysis and wrote the manuscript. TY contributed to the design of the study and data collection. JZ revised this manuscript. All authors contributed to the article and approved the submitted version.

## Funding

This work was supported by the National Office for Philosophy and Social Sciences [CHA 210263].

## Conflict of interest

The authors declare that the research was conducted in the absence of any commercial or financial relationships that could be construed as a potential conflict of interest.

## Publisher’s note

All claims expressed in this article are solely those of the authors and do not necessarily represent those of their affiliated organizations, or those of the publisher, the editors and the reviewers. Any product that may be evaluated in this article, or claim that may be made by its manufacturer, is not guaranteed or endorsed by the publisher.
